# Temporomandibular disorder and somatic symptoms: Relations to ‘fear of missing out’ and other negative emotional states

**DOI:** 10.2340/aos.v83.40776

**Published:** 2024-05-28

**Authors:** Adrian Ujin Yap, Florencia Kurniawan, Yenny Pragustine, Carolina Marpaung

**Affiliations:** a Department of Dentistry, Ng Teng Fong General Hospital and Faculty of Dentistry, National University Health System, Singapore, Singapore; b National Dental Research Institute Singapore, National Dental Center Singapore and Duke-NUS Medical School, Singapore Health Services, Singapore, Singapore; c Department of Prosthodontics, Faculty of Dentistry, Universitas Trisakti, Jakarta, Indonesia

**Keywords:** Temporomandibular joint disorders, Somatization, Depression, Anxiety, Stress

## Abstract

**Objective:**

The association between the ‘fear of missing out (FOMO)’ and physical symptoms has not been widely explored. This study aimed to investigate the relationship between FOMO and other negative emotions with Temporomandibular disorder (TMD) and somatic symptoms in young adults. The correlations between the various physical and emotional variables were also established.

**Material and methods:**

TMD and somatic symptoms were appraised with the Short-form Fonseca Anamnestic Index, quintessential five TMD symptoms of the Diagnostic Criteria (DC)/TMD, and Patient Health Questionnaire-15. FOMO and other negative emotional states were assessed with the FOMO Scale and Depression, Anxiety, Stress Scales-21 (DASS-21). Data were evaluated using non-parametric tests/correlation and regression analysis (α = 0.05).

**Results:**

While only negative affectivity (total DASS), anxiety, and stress differed significantly between those without and with TMDs, significant variances in FOMO and all DASS-21 constructs were discerned between individuals without and with somatization.

**Conclusions:**

Individuals with orofacial pain and more severe somatic symptoms have higher levels of negative emotions including FOMO. While somatization increased the prospect of TMDs, being female, presence of TMDs, and negative affectivity were risk factors for somatization in young adults.

**Clinical relevance:**

Asian young adults appear to be disposed to somatization, and TMDs may be a form of functional somatic syndromes. Recognition of somatic symptoms and emotional distress, including FOMO, is essential for person-centric TMD care.

## Introduction

‘Fear of missing out (FOMO)’, a contemporary negative emotional construct, has been defined as the ‘pervasive apprehension that others might be having rewarding experiences from which one is absent’ [1]. It is experienced by many young people and is characterized by the persistent desire to stay continuously connected with others in one’s social network [1, 2]. FOMO is posited to be a predisposing factor for excessive internet and social media use [3–6]. More specifically, the problematic use of social networking sites (SNSs) accompanying FOMO is contributed by the ease of ‘at-will’ interactions offered by modern mobile devices and the constant need for personal validation and rewarding experiences [6]. The latter is manifested by compulsive behaviors to maintain social connections, such as the frequent checking and engagement of SNSs, which could heighten anxiety levels. Furthermore, the continuous ‘upward social comparisons’ and ‘unreasonable expectations’ created can bring about poor self-esteem as well as depression [7]. FOMO did not diminish during the COVID-19 pandemic, even when socially distanced at home, but instead strengthened attitudes toward online communications and relational closeness [8, 9]. It has been associated with a range of adverse physical and psychological effects including sleep deprivation, reduced life competency, a lack of emotional control, and emotional distress [6, 10]. While the link between FOMO and other negative emotional states was explored in recent years [4, 10], there is a paucity of research regarding its relation to somatic symptoms including Temporomandibular disorders (TMDs). The latter is clinically important given the known relationship of somatic and TMD symptoms to depression and anxiety, which could be mediated by FOMO in young people [11–13].

East and Southeast Asians have a greater propensity for somatization (the manifestation of psycho-emotional distress through somatic symptoms) than Westerners [14, 15]. This phenomenon had been attributed to the stigma, interpersonal sensitivity, low social support, and rates of treatment-seeking associated with mental illness, as well as the overt emphasis on ‘somatic idioms’ of distress in Asian cultures [13, 15–17]. The ‘persistent and troublesome’ somatic complaints entail one or more organ or physiological (musculoskeletal, nervous, cardio-respiratory, gastrointestinal, and/or genito-urinary) systems and can be clustered into functional somatic disorders that encompass fibromyalgia, chronic fatigue, irritable bowel, and other syndromes [18].

TMDs are a heterogeneous group of musculoskeletal conditions characterized by pain and/or dysfunction of the stomatognathic system [19]. They affect up to 16% of the general population and share many epidemiological and etiopathogenetic features with functional somatic syndromes [19, 20]. As such, TMDs have been postulated to be a form of functional somatic syndromes with central sensitization (amplified response of the central nervous system to peripheral inputs) being the coalescing factor [21]. This was corroborated by the high prevalence of moderate-to-severe somatization (up to 77%) in TMD patients [11]. More recently, somatic symptoms and stress were found to predict TMDs, whereas TMD symptoms, stress, and being female were risk factors for somatic symptoms in Asian community youths [13]. Additionally, somatic, depressive, and anxiety symptoms were observed to be related to TMDs in young people, even when not severe [22].

Considering the aforesaid and the recognition of FOMO as a modern psychological phenomenon [1, 6], this study aimed to investigate the associations of TMD and somatic symptoms to FOMO and other negative emotional states in Asian young adults. Correlations between TMD, somatic, FOMO, depressive, anxiety, and stress symptoms were also examined. The research hypotheses were as follows: (1) young adults with TMDs and somatization experience greater levels of FOMO and emotional distress, (2) the type of TMD symptoms and degree of somatic symptoms are related to the extent of FOMO and emotional distress, and (3) TMD, somatic, negative affectivity, depression, anxiety, and stress scores are correlated.

## Materials and methods

### Study design and sample

The study was authorized by the ethics committee of the Faculty of Dentistry, Universitas Trisakti, Indonesia (reference number: 013/S3/KEPK/FKG/9/2021). Young adults were recruited from a university in the capital city of Indonesia (Jakarta) using a voluntary sampling procedure. The inclusion criteria were individuals aged 18 to 24 and English proficiency. Those with prior orofacial trauma or receiving medical care for debilitating physical and/or psychiatric conditions were duly excluded. A minimum of 205 participants was determined with an online calculator (https://www.calculator.net/sample-size-calculator.html) based on a 95% confidence level, 5% margin of error, 16% prevalence of TMDs,^19^ and enrollment of 20,638 students. All potential study participants were provided information on the study and informed consent was obtained before administering an online survey containing demographic details, the Short-form Fonseca Anamnestic Index (SFAI), quintessential five TMD symptoms (5Ts) of the Diagnostic Criteria for TMDs Symptom Questionnaire (DC/TMD-SQ), Patient Health Questionnaire-15 (PHQ-15), FOMO Scale, and Depression, Anxiety, Stress Scales-21 (DASS-21) [1, 23–26].

### Study measures

#### TMD and somatic symptoms

The presence of TMDs was ascertained with the 5-item SFAI, which was developed to reduce the dimensionality and improve the diagnostic accuracy of the parent instrument [23]. The SFAI has good psychometric properties and high specificity and sensitivity when referenced to both the Research Diagnostic Criteria for TMDs (RDC/TMD) and DC/TMD standards [23, 27, 28]. Items are scored using a 3-point response scale with ‘no’ = 0 points, ‘sometimes’ = 5 points, and ‘yes’ = 10 points. Total SFAI scores ≥15 points specify that TMDs are present [23]. To differentiate the influence of TMD pain and dysfunction, the archetypal five TMD symptoms (5Ts) were appraised with the key items from the DC/TMD-SQ which had ‘no’ and ‘yes’ response options. The latter were assigned 0 and 10 points accordingly to follow the SFAI scoring scheme and facilitate ensuing correlation analysis. Participants were subsequently categorized into no, pain-related (TMD/facial pain and headache), intra-articular (Temporo-Mandibular Joint (TMJ) sounds, closed, and open-locking), and mixed (both pain-related and intra-articular) TMD groups [24, 29]. The 5Ts were recently demonstrated to exhibit high accuracy for distinguishing both pain-related and/or intra-articular disorders [24]. The presence of somatization and somatic symptoms severity was assessed with the PHQ-15 [25]. The reliability and validity of the PHQ-15 are well established and it has been utilized in a variety of healthcare settings as well as the Axis II of the DC/TMD [25, 29, 30]. The items, which included the most prevalent Diagnostic and Statistical Manual of Mental Disorders DSM-IV-TR somatization disorder somatic symptoms [31], are rated using a 3-point response scale with ‘not bothered at all’ = 0 points, ‘bothered a little’ = 1 point, and ‘bothered a lot’ = 2 points. Total PHQ-15 scores span from 0 to 30 points with scores of ≥5 points indicating the presence of somatization and 5, 10, and 15 points serving as the cut-points for low, medium, and high somatic symptoms severity respectively.

#### FOMO and negative emotional states

FOMO was evaluated with the 10-item FOMO Scale that deals with the ‘angst that others are having fun without you’ [1]. It is the first experimentally authenticated measure of FOMO and had been widely used in psychological research and validated in the target population [32, 33]. Items are scored on a 5-point response scale ranging from ‘no at all true of me’ = 1 point to ‘extremely true of me’ = 5 points. Total FOMO scores vary between 10 and 50 points with greater scores specifying higher levels of FOMO. The negative emotional states of depression, anxiety, and stress were assessed with the DASS-21 which contained 7 items for each construct [26]. The DASS-21 has good measurement properties and has been found to have a bifactor structure consisting of a general factor for negative affectivity (personality trait referring to the disposition to experience negative emotions and poor self-concept) and the different subscales [34]. The items are rated on a 4-point response scale extending from ‘did not apply to me at all’ = 0 points to ‘applied to me very much or most of the time’ = 3 points. Total DASS scores, which denote negative affectivity, range from 0 to 63 points, while subscale scores vary between 0 and 21 points. Greater scores signify higher levels of negative affectivity, depression, anxiety, and stress symptoms. Cut-points for the severity categorization (normal to extremely severe) for the three subscales are indicated in the DASS manual [26].

### Statistical analyses

Statistical evaluations were conducted using the Statistical Package for the Social Sciences software version 27.0 (IBM Corporation, Armonk, New York, USA) with a significance level of 0.05. Categorical data were presented as frequencies with percentages and examined with the Chi-square test. Numerical data were reported as means with standard deviations (SD) as well as medians with inter-quartile ranges (IQR) and subjected to normality testing using the Shapiro-Wilk’s test. As numerical data were not normally distributed, Kruskal-Wallis and post-hoc Mann-Whitney U tests were applied. Relations between SFAI, 5Ts, PHQ-15, FOMO, and DASS-21 scores were examined with Spearman’s rank-order correlation with coefficients (*r_s_*) of 0.1, 0.4, and 0.7 serving as cut-points for weak, moderate, and strong associations accordingly [35]. Multivariate logistic regression analysis was carried out to explore the physical and emotional predictors for the presence of TMDs and somatization. A stepwise variable selection process was used with a threshold of *p* < 0.10 for eliminating insignificant variables. Outcomes were presented as odds ratios (ORs) with 95% confidence intervals (95% CIs).

## Results

### Descriptive data

Out of the 423 young adults who responded to the call for study participation, 8 met the exclusion criteria and none returned incomplete surveys. The final study sample (*n* = 415) had a mean age of 22.7 ± 1.1 years and 85.3% were women. Among the participants, 18.3% and 60.7% reported the presence of TMDs and somatization respectively (Table 1). The age of the participants without and with TMDs/somatization was similar. While the proportion of women without (85.0%) and with (86.8%) TMDs was comparable, the percentage of female participants without (76.7%) and with (90.9%) somatization differed substantially.

**Table 1:** Sociodemographic characteristics of the study sample (n = 415).

Variables	*n* (%)	Age			Gender		
		Mean (SD)	Median (IQR)	p*	Male *n* (%)	Female *n* (%)	*p*^
							
**Total**	415 (100.0)	22.7 (1.1)	23.0 (2)	-	61 (14.7)	354 (85.3)	-
							
**TMDs (SFAI)**							
Absent	339 (81.7)	22.7 (1.1)	23.0 (2)	0.414	51 (15.0)	288 (85.0)	0.068
Present	76 (18.3)	22.6 (1.1)	23.0 (2)		10 (13.2)	66 (86.8)	
							
**Somatization (PHQ-15)**							
Absent	163 (39.3)	22.8 (1.1)	23.0 (2)	0.225	38 (23.3)	125 (76.7)	**< 0.001**
Present	252 (60.7)	22.6 (1.1)	23.0 (2)		23 (9.1)	229 (90.9)	

SFAI: Short-form Fonseca Anamnestic Index; PHQ-15: Patient Health Questionnaire-15; SD: Standard deviation; IQR: Inter-quartile range. Results of Mann-Whitney U* and Chi-square° tests. Bold indicates *p* < 0.05.

#### FOMO and emotional distress

Table 2 shows the mean/median FOMO and DASS-21 scores for participants without (absent) and with (present) TMDs/somatization. While no significant differences in FOMO and depression scores were observed, participants with TMDs exhibited significantly higher total DASS, anxiety, and stress scores than their peers without TMDs. However, significant differences in FOMO, total DASS, depression, anxiety, and stress scores were observed between those without and with somatization. Table 3 displays the mean/median FOMO and DASS-21 scores of the participants when categorized by TMD subtypes (no [NT], pain-related [PT], intra-articular [IT], and mixed [MT] TMD symptoms) and somatic symptoms severity (normal [NS], low [LS], medium [MS], and high [HS] somatic symptoms severity). Participants with painful (PT and MT) TMD and more severe somatic symptoms had higher levels of negative emotions including FOMO. Significant differences in FOMO (PT > NT), total DASS (MT > NT), anxiety (MT > IT and NT), and stress (MT > NT) were noted among the various TMD subtypes. Significant differences in FOMO, total DASS, depression, anxiety, and stress scores were observed among the different somatization groups (HS, MS, LS > NS). Additionally, the HS group also presented significantly greater total and all subscale DASS scores than the LS group.

**Table 2:** Mean/median FOMO, total DASS (negative affectivity), depression, anxiety, and stress scores for participants without and with TMDs/somatization.

Variables	TMDs (SFAI)			Somatization (PHQ-15)		
	Absent	Present	p*	Absent	Present	p*
*n* **(%)**	339 (81.7)	76 (18.3)	-	163 (39.3)	252 (60.7)	-
**FOMO**						
Mean (SD)	20.5 (7.3)	21.7 (7.8)	0.134	19.2 (6.9)	22.5 (7.8)	**< 0.001**
Median (IQR)	20.0 (9)	21.0 (12)		18.0 (9)	21.5 (12)	
**Total DASS**						
Mean (SD)	13.16 (9.6)	15.3 (10.5)	**0.037**	9.8 (8.3)	17.4 (10.2)	**< 0.001**
Median (IQR)	12.0 (14)	14.0 (12)		8.0 (12)	15.5 (14)	
**Depression**						
Mean (SD)	3.2 (3.6)	3.4 (3.5)	0.203	2.2 (2.9)	4.0 (3.7)	**< 0.001**
Median (IQR)	2.0 (5)	2.0 (4)		1.0 (3)	3.0 (5)	
**Anxiety**						
Mean (SD)	4.1 (3.1)	5.0 (3.6)	**0.008**	3.1 (2.6)	5.6 (3.6)	**< 0.001**
Median (IQR)	4.0 (5)	4.0 (5)		3.0 (4)	5.0 (5)	
**Stress**						
Mean (SD)	5.9 (4.0)	6.9 (4.5)	**0.034**	4.6 (3.9)	7.7 (4.2)	**< 0.001**
Median (IQR)	6.0 (5)	6.0 (7)		4.0 (6)	7.0 (5)	

TMD: Temporomandibular disorder; SFAI: Short-form Fonseca Anamnestic Index; PHQ-15: Patient Health Questionnaire-15; FOMO: Fear of missing out; DASS: Depression, Anxiety, Stress Scales-21; SD: Standard deviation; IQR: Inter-quartile range. Results on Mann-Whitney U test*. Bold indicates *p* < 0.05.

**Table 3:** Mean/median FOMO, total DASS (negative affectivity), depression, anxiety, and stress scores for the various TMD subtypes and somatic symptom severity.

Variables	TMD subtypes					
	No TMD (NT)	Pain-related TMD (PT)	Intra-articular TMD (IT)	Mixed TMD (MT)	p*	*Post-hoc*
*n* **(%)**	171 (41.2)	72 (17.3)	88 (21.2)	84 (20.2)	-	-
**FOMO**						
Mean (SD)	20.3 (7,7)	22.9 (7.6)	21.1 (7.3)	21.8 (7.7)	**0.049**	PT > NT
Median (IQR)	20.0 (11)	23.0 (13)	21.0 (11)	20.5 (10)		
**Total DASS**						
Mean (SD)	13.1 ( 10.3 )	15.7 (9.8)	13.3 (9.6)	17.0 (10.2)	**0.005**	MT > NT
Median (IQR)	12.0 (14)	15.0 (13)	12.5 (13)	15.5 (14)		
**Depression**						
Mean (SD)	3.2 ( 3.5 )	3.4 (3.5)	3.1 (3.2)	3.7 (3.8)	0.572	-
Median (IQR)	2.0 (5)	2.0 (5)	2.0 (4)	2.5 (4)		
**Anxiety**						
Mean (SD)	4.0 ( 3.3 )	5.1 (3.3)	4.2 (3.5)	5.9 (3.4)	**< 0.001**	MT > IT, NT
Median (IQR)	4.0 (5)	5.0 (4)	3.0 (5)	5.0 (4)		
**Stress**						
Mean (SD)	5.9 (4.4)	7.2 (4.3)	6.0 (4.2)	7.4 (4.1)	**0.011**	MT > NT
Median (IQR)	6.0 (5)	7.0 (6)	6.0 (7)	7.0 (7)		
Variables	Somatic symptoms severity					
	Normal (NS)	Low (LS)	Medium (MS)	High (HS)	p*	*Post-hoc*
*n* **(%)**	163 (39.3)	130 (31.3)	67 (16.1)	55 (13.3)	-	-
**FOMO**						
Mean (SD)	19.2 (6.8)	21.9 (7.4)	22.7 (8.0)	23.7 (8.6)	**< 0.001**	HS, MS, LS > NS
Median (IQR)	18.0 (9)	21.0 (9)	21.0 (13)	24.0 (14)		
**Total DASS**						
Mean (SD)	9.8 (8.3)	15.2 (9.7)	17.1 (8.6)	22.8 (11.1)	**< 0.001**	HS, MS, LS > NS HS > LS
Median (IQR)	8.0 (12)	13.0 (11)	16.0 (13)	21.0 (16)		
**Depression**						
Mean (SD)	2.2 (2.9)	3.5 (3.5)	3.7 (3.2)	5.5 (4.3)	**< 0.001**	HS, MS, LS > NS HS > LS
Median (IQR)	1.0 (3)	2.0 (4)	3.0 (5)	4.0 (6)		
**Anxiety**						
Mean (SD)	3.1 (2.6)	4.8 (3.3)	5.5 (3.3)	7.8 (3.7)	**< 0.001**	HS, MS, LS > NS HS, MS > LS
Median (IQR)	3.0 (4)	4.0 (4)	5.0 (4)	8.0 (5)		
**Stress**						
Mean (SD)	4.6 (3.9)	6.9 (4.0)	7.9 (3.8)	9.5 (4.3)	**< 0.001**	HS, MS, LS > NS HS > LS
Median (IQR)	4.0 (6)	6.0 (5)	8.0 (5)	9.0 (6)		

FOMO: Fear of missing out; DASS: Depression, Anxiety, Stress Scales-21; SD: Standard deviation; IQR: Inter-quartile range. Results of Kruskal-Wallis/post-hoc Mann-Whitney U tests*. Bold indicates *p* < 0.05.

### Correlations and regression analysis

Table 4 indicates the correlation coefficients between the various physical and emotional variables. Though associations were generally significant, correlation coefficients fluctuated from negligible to strong. While moderately strong correlations were detected between SFAI and 5Ts scores (*r_s_* = 0.69), their relationships to PHQ-15, FOMO, and DASS-21 scores were mostly weak (*r_s_* = 0.08–0.39). Although PHQ-15 scores were weakly correlated to FOMO and depression scores (*r_s_* = 0.24–0.34), their correlations to total DASS, anxiety, and stress were moderately strong (*r_s_* = 0.44–0.46). Moderately strong correlations were also discerned between FOMO and total DASS/stress scores (*r_s_* = 0.40). Furthermore, moderately strong to strong relationships were observed between total DASS, depression, anxiety, and stress subscales (*r_s_* = 0.60–0.95).

**Table 4:** Correlations between SFAI, 5Ts, PHQ-15, FOMO, total-DASS, depression, anxiety, and stress scores.

	SFAI	5Ts	PHQ-15	FOMO	Total DASS	Depression	Anxiety
SFAI	-	-	-	-	-	-	-
5Ts	0.69**	-	-	-	-	-	-
PHQ-15	0.27**	0.39**	-	-	-	-	-
FOMO	0.08	0.11*	0.24**	-	-	-	-
Total DASS	0.13**	0.16**	0.46**	0.40**	-	-	-
Depression	0.08	0.08	0.34**	0.29**	0.84**	-	-
Anxiety	0.18**	0.22**	0.46**	0.36**	0.88**	0.60**	-
Stress	0.12*	0.15**	0.44**	0.40**	0.95**	0.71**	0.80**

TMD: Temporomandibular disorder; SFAI: Short-form Fonseca Anamnestic Index; 5Ts: five TMD symptoms; PHQ-15: Patient Health Questionnaire-15; FOMO: Fear of missing out; DASS: Depression, Anxiety, Stress Scales-21. Results of Spearman’s correlation.

* indicates *p* < 0.05,

** indicates *p* < 0.01.

The outcomes of multivariate logistic regression analysis are reflected in Table 5. The prospect of TMDs was influenced by the presence of somatization (OR = 2.11; 95% CI = 1.41–3.16). The likelihood of somatization was predicted by the female gender (OR = 2.97; 95% CI = 1.60–5.60), presence of TMDs (OR = 1.88; 95% CI = 1.21–2.92), and negative affectivity (OR = 1.09; 95% CI = 1.06–1.12).

**Table 5:** Results of multivariate logistic regression analyses for the presence of TMDs and somatization.

Variable	Presence of TMDs		Presence of Somatization	
	Odds Ratio (95% CI)	p*	Odds Ratio (95% CI)	p*
Gender				
Male	Reference	-	Reference	
Female			2.97 (1.60–5.60)	**0.001**
Somatization present	2.11 (1.41–3.16)	**< 0.001**		
TMD present	-	-	1.88 (1.21–2.92)	**< 0.001**
FOMO				
Negative affectivity			1.09 (1.06–1.12)	**<0.001**
Depression				
Anxiety				
Stress				

FOMO: fear of missing out; TMD: Temporomandibular disorder.

Results of multivariate logistic regression analysis*. Bold indicates *p* < 0.05.

## Discussion

This is the first study to relate TMD and somatic symptoms to FOMO and other negative emotional constructs in Asian young adults. The study is pertinent given the pervasiveness of FOMO in modern society and its possible diverse physical and/or psychological consequences [1, 6]. The first two research hypotheses were supported as FOMO/emotional distress levels varied with the presence of TMDs/somatization and were influenced by TMD subtypes/somatic symptoms severity. The third research hypothesis was also endorsed as significant correlations, albeit weak, were observed between the various physical and emotional variables. Young adults were identified for this study as TMDs, somatization, and FOMO are common in this age group and often associated with the development of other psychological problems [1, 19, 36]. University students, in particular, are subjected to high levels of academic, societal, and life stresses, and high rates of emotional distress, which may be compounded by FOMO, had been reported among them [37]. The prevalence of TMDs based on the SFAI (18.3%) was comparable to that reported for the general population when determined with formalized diagnostic criteria (15.8%) [19]. The occurrence of somatization (60.7%) was consistent with that of other Southeast Asian youths (65.0%) when assessed with the PHQ-15 [13]. Medium-to-high levels of somatic symptoms were found in 29.4% of the participants which was 1.5 folds greater than the prevalence rates observed in primary care patients (18.5%) [38]. Findings corroborated earlier work indicating the tendency of Asians to somatize [14, 15].

### TMDs and negative emotions

The association between TMDs and negative emotions had been demonstrated in both non-clinical and clinical samples [13, 39–42]. While depression (feeling of despair/despondence), anxiety (feeling of apprehension/worry), and stress (body’s response to tension/threats) had been implicated in TMDs, depression appears to play a larger role in TMD patients, especially when chronic pain is involved [41]. In the present study, only negative affectivity, anxiety, and stress differed significantly between participants without and with TMDs. Findings were consistent with prior research on Asian community youths and can be contributed by the mostly normal levels of depression in the study samples [13, 40]. The weak correlations between TMD and anxiety/stress scores (*r_s_* = 0.12–0.22) observed could also be ascribed to lower extents of anxiety and stress experienced when compared to TMD patients [41, 42].

Though FOMO was not associated with the presence of TMDs as determined by the SFAI, individuals with pain-related TMD symptoms presented significantly higher levels of FOMO than those with no TMD symptoms. The disparity could be attributed to the inclusion of headaches in the 5Ts which were discarded during the SFAI development [43]. Headaches affect about 46% of adults and explain the higher proportion of participants with TMD symptoms based on the 5Ts [44]. Besides TMDs, headaches can be caused by many other medical conditions and are often triggered by stress which was moderately correlated to FOMO (*r_s_* = 0.40). The significantly higher levels of negative affectivity, anxiety, and stress reported by the MT group than the NT/IT groups were anticipated as they encountered both pain and function-related TMD problems that could reduce life quality considerably [41].

After adjusting for possible confounders, only somatization was found to increase the risk of TMDs. More specifically, it doubled the probability of TMDs in the study sample. This together with studies specifying the high occurrences of somatization and other comorbid chronic pain conditions among TMD patients lend support to the belief that TMDs are a form of functional somatic syndromes [11, 20, 21, 45].

### Somatization and negative emotions

FOMO, negative affectivity, depression, anxiety, and stress levels varied significantly between participants without and with somatization as well as among the various somatic symptom severity groupings. Findings were foreseen as somatization is essentially the somatic expression of psycho-emotional distress. Although scores for all negative emotional constructs including FOMO escalated with increasing somatic symptoms severity, moderately strong correlations to PHQ-15 scores were perceived just for negative affectivity, anxiety, and stress (*r_s_* = 0.44–0.46). The depression, anxiety, and stress subscales were all strongly related to negative affectivity (*r_s_* = 0.84–0.95) validating the bifactor structure of the DASS-21 where each subscale taps on the general dimension of distress [34, 46]. While depression scores were in the normal to mild range (0–6 points), the high somatic symptoms group presented severe anxiety (8–9 points) and moderate stress (9–10 points) [26]. The strong correlation between anxiety and stress (*r_s_* = 0.80) supports the notion that anxiety is the body’s reaction to stress. The two emotional constructs share common behavioral and neural processes ensuing in identical physical symptoms including muscle pain, insomnia, and fatigue [47]. FOMO was also moderately associated with stress (*r_s_* = 0.40) and corroborated the recent work of Yang et al. suggesting that FOMO acted as the mediator between problematic smartphone use and stress [48].

Multivariate regression analysis indicated that being female tripled the prospect of somatization. Furthermore, the presence of TMDs and negative affectivity increased its probability by 88% and 9% respectively. Women are known to report more numerous, frequent, and intense bodily symptoms, including TMDs, than men [49, 50]. Many contributing factors had been implicated including socio-cultural and hormonal issues as well as gender differences in somatic/visceral processing, the incidence of abuse/violence, and prevalence of depression/anxiety [49, 50]. The relationship between somatic and TMD symptoms was deliberated earlier and explained by central sensitization. Though its underlying mechanisms are not fully understood, central sensitization is thought to be caused by decreased inhibitory synaptic transmission, increased excitatory synaptic transmission, and is induced by proinflammatory cytokines [51]. Central sensitization helps clarify the comorbidities of TMDs and other functional somatic syndromes and also applies to arthritic pain and complex regional pain syndromes [52]. The likelihood of somatization was increased only marginally by negative affectivity, the general factor for psychological distress, and could be contributed by the relatively lower levels of depression, anxiety, and stress among community samples when contrasted to TMD patient populations [41, 42]. Findings were congruous with that of Yap et al. who determined that somatization was predicted by the female gender, TMDs, and stress in other Southeast Asian youths [13]. Though negative affectivity was not specifically assessed in their study, similar outcomes are expected given the very strong correlation between total DASS and stress (*r_s_* = 0.95) found in this study. As there is strong evidence supporting the bifactor structure of the DASS-21, future research would benefit from the incorporation of negative affectivity (total DASS) into their assessment [34, 46].

### Study limitations

This observational study has some limitations. First, causal relationships between the negative emotional states and physical symptoms cannot be established due to the cross-sectional design employed. Causality can only be inferred in observational research utilizing prospective cohort and nested case-control designs [53]. Second, not all young adults in the country were represented by the study sample. Moreover, women were predominant among the study participants. The latter can be explained by the greater inclination of women to contribute to online surveys and the voluntary sampling method applied [54]. Future work should ideally encompass more male participants, unemployed as well as working young adults. The study could also be extended to TMD patients, in addition to other racial and ethnic groups. Lastly, the use of self-reported data may subject the study to information bias including recall, social desirability, as well as measurement partialities [55].

## Conclusions

Among the young adults examined, 18.3% and 60.7% reported the presence of TMDs and somatization, respectively. While only negative affectivity, anxiety, and stress differed significantly between participants without and with TMDs, significant variances in all negative emotional constructs including FOMO were discerned between those without and with somatization. Individuals with painful TMD and more severe somatic symptoms had higher levels of negative emotions. Nevertheless, only somatic symptoms and FOMO were moderately correlated to negative affectivity and stress. While somatization increased the prospect of TMDs, being female, the presence of TMDs, and negative affectivity were risk factors for somatization. Collectively, the findings suggest that Asian young adults are disposed to somatic symptoms, and TMDs may be a form of functional somatic syndromes. Recognition of somatization and emotional distress including FOMO is thus essential for person-centric TMD care.
